# The Different Levels of Quantum: A Methodological–Philosophical Portrait of Computational Quantum Chemistry

**DOI:** 10.1021/acsphyschemau.6c00097

**Published:** 2026-08-10

**Authors:** Sindy Julieth Rodríguez-Sotelo, Sebastian Kozuch

**Affiliations:** Ben Gurion University of the Negev, Beer-Sheva 841051, Israel

**Keywords:** tunneling, quantum levels, *in silico* versus *in vitro*, computational chemistry, epistemology of chemistry

## Abstract

*In silico* and *in vitro* experimental chemistry do have many similarities in terms of following the scientific method, but they also have epistemological differences. Maybe the most striking difference is that wet chemistry, in terms of physical effects, is all-inclusive; it is not possible to remove relativistic effects or the zero-point energy (to give a couple of examples) from the molecules in a real-life flask. On the contrary, computational chemistry requires a conscious and careful decision on whether these specific effects should be included in the virtual experiment. Omit them, and the chemistry will not be realistic; incorporate unnecessary effects, and the job may be intractable. In this perspective, we will describe one direction to add quantum effects, depending on the reach and requirements of the studied question. From the electronic to the nuclear wave function and beyond, the addition of deeper and deeper quantum effects must be sequentially and judiciously nested as a matryoshka doll.

## Graphical abstract

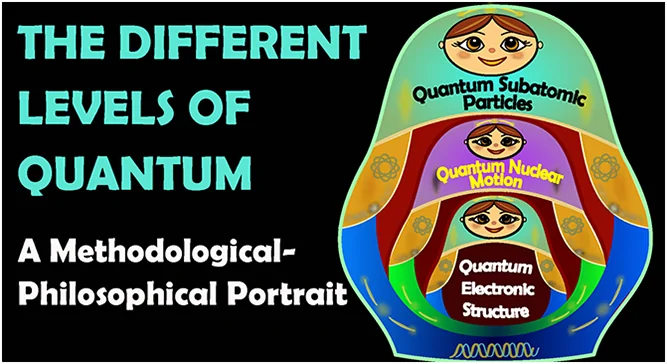

## 1 Philosophical Introduction

This article delves into the different levels of quantum computational chemistry. When pondering what to include, we weighed whether we should take a methodological direction, or dive into a more philosophical analysis. Of course we ended up mixing both approaches, since one strengthens the other. This risked creating tedious technical writing for the more philosophically inclined (as a reviewer rightfully indicated), or alienating the more “hands-on” public with abstract diatribes. Therefore, we recommend the reader skim over the personally unappealing text, and focus on their most subjectively interesting parts (or, as it was said, “Research your own experience; absorb what is useful, reject what is useless and add what is essentially your own”^1^).

### 1.1 Computation Is Experimentation

Typically, the opposite of computational chemistry is considered to be experimental chemistry. Such a distinction is not only untrue, but for our community it is also, to be frank, impolite. Computational chemistry is experimentation, and as such, it is experimental chemistry. It is proposing hypotheses, testing them, and proving them right (or falsifying them to prove them wrong). The full scientific method. As such, instead of terming them as computational versus experimental, we may distinguish them as virtual vs material chemistry, or, as it is sometimes said, *in silico* vs *in vitro*. To emphasize the distinction, we can talk about “real-flask”, “real-life”, or “real-world” experiments, but never about “genuine”, “authentic”, or “real” experiments against computational ones. And remember that if you carry out experiments, it follows that you work in a laboratory, not in an office, even if you do not wear safety glasses.

Every computational chemist is partly a theoretician, but the same might be said about material chemists. *In silico* chemistry relies on models and approximations, but so do the interpretations of *in vitro* research. In the wet lab most experiments do not work, but the same happens in the dry lab. Undoubtedly, the computer is a complete experimental environment to practice chemistry (Figure 1).

**Figure 1:**
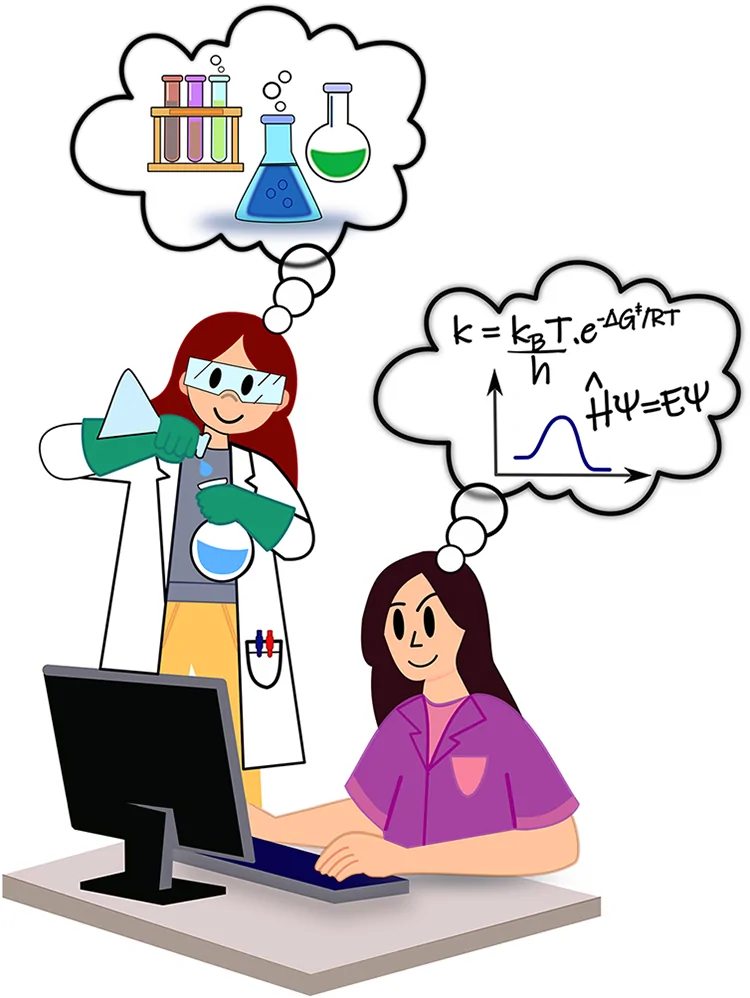
*In vitro* vs *in silico*, instrumental vs algorithmic data. Both are experimental chemistry, even when using different technical and philosophical approaches to obtain and analyze the results.

Still, there are clear contrasts (beyond the fact that we, computational chemists, do not get our hands dirty).

### 1.2 On the Epistemology of Computational Chemistry

First, and evidently, there is the issue of the ontology.^2^ Simply put, are the experiments and results obtained with computational chemistry real or not? On a first shallow thought, we may say that it is not, since virtual reality is, by nature, the exact opposite of real life. On the other hand, some philosophers, especially Plato, debated that the realm of the Forms or Ideas is the essence of everything, and matter barely reproduces those ideas (the “realistic” and “idealistic” philosophical positions). We may discuss accordingly if a computed mechanism of a chemical reaction, aromaticity, or a molecular orbital are phenomena (an observable event) or noumena (a thing in itself).^2,3^ We will leave this debate to philosophers, but the essence of this question should hover over every chemist.

In contrast, the question of interest of this manuscript will be epistemological.^2^ Simply put, how do we know what we know, in the virtual lab?

One of the most critical philosophical and practical differences between both types of laboratories resides in the source of the information. In *in vitro* chemistry we can only obtain the final outcome of the experiment (yield, rate, exothermicity, crystal structure, spectra, etc.), which includes each and every possible existing effect. It is impossible to filter out some specific parameters to cleanly obtain a desired one. For instance, we cannot turn on and off relativistic effects, or study *in situ* an individual intermediate within an active, running catalytic reaction. We can obviously minimize effects with the *ceteris paribus* approach (“all other things being equal”). This can be achieved for instance in classical thermodynamics by changing the pressure while keeping the temperature fixed or vice versa. Another example in more modern physical organic chemistry involves the linear-free energies relationships (LFERs), which can distinguish between orthogonal effects; for instance in the Swain Lupton equation the field and resonant effects are set apart, or in the Taft equation the steric and polar effects are discriminated. And evidently we can cover the flask to avoid photophysical effects, cool down the equipment to avoid thermal effects, or avoid adding a reactant to stop a catalytic reaction midcycle. But to avoid the zero-point energy can be much trickier (isotopic substitutions might do the trick, up to a certain point). The point is, in material chemistry all the considered and not-considered effects are included, like it or not. Only with special, ingenious experiments it may be possible to individualize some of those effects, but only if we already know that they exist!

In many (if not most) of the “real-life” experiments we can obtain the “what”, but cannot fully grasp the “why”. That is, we can certainly get the final result of the experiment without understanding its chemical reasons. This is a substantial disadvantage; however, the advantage is much more significant: the experiment will depict the reality.

The world of computational chemistry exhibits a striking contrast in this sense. Through virtually observing the molecules and the reactions, we can, ideally and in theory, see the occurring processes *in situ* in our screens. But we can never be completely sure that the obtained final result will truly match the real-world one. Paraphrasing the famous saying, it is difficult to make predictions, especially about the chemical reality.^4–6^ Without the feedback of material chemists and their *in vitro* experimental results, most of our keytional expectations will not hit the target. Essentially, we can propose the “why” and the “how”, but we cannot be certain of the “what” (the actual experimental outcome) without validation. This is a substantial disadvantage; however, the advantage is much more significant: the *in silico* experiment can explain and enlighten.

An example of the contribution of each approach, and the benefits of their synergism, can be observed in the mechanistic study of a benzoin condensation, recently carried out between our laboratory and Milo’s group (we apologize for providing examples from our own lab, but, evidently, they are the cases we know the best).^7^ As shown in Figure 2, the “textbook” mechanism of the reaction is relatively simple, almost deceivingly straightforward. This was deduced from experimental observables, basically from which species are produced and consumed, and with which order of reaction. But, as a general rule, and without any intention of causing offense, whatever mechanism material chemists propose, it will necessarily be an oversimplification, at best. There is a limit on the amount of details that instrumental equipment can provide. In parallel, the computed cycle of Figure 2 shows a much more detailed and rich chemistry, with more than twice the number of steps, and a better chance to detect the determining states. However, considering the infinite reactivity possibilities, there would be virtually no chance that computational chemistry would converge to the real product without some real-lab inputs in terms of detected species (although current work on automated mechanism prediction and machine learning may bring some hope in this research direction).^8–11^ This is the essential difference between the “what” and the “why”.

**Figure 2:**
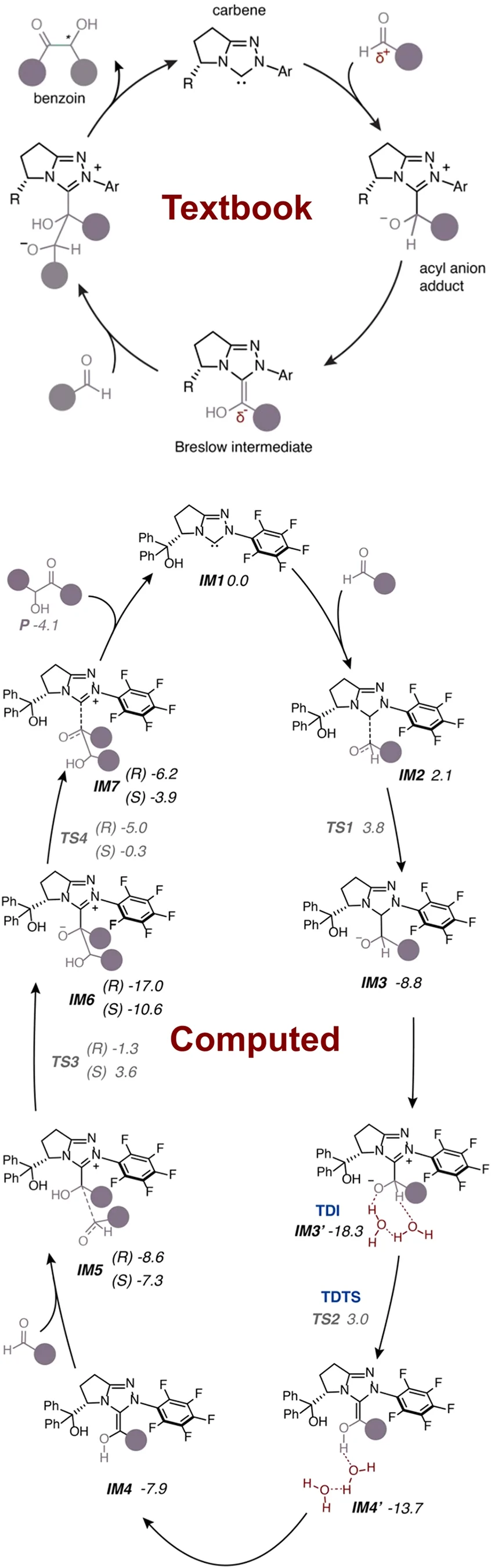
Textbook vs computed benzoin condensation. Adapted with permission from ref 7. Copyright 2023 American Chemical Society.

At the center of this computational conundrum resides the fact that we can know exactly what concepts are poured into our computations, in what we can call the “known knowns”. If we are well-educated in the field, we will wisely neglect other concepts that will be irrelevant to the experiment at hand; we can call them the “known unknowns”,^12^ the theoretical terms we know to exist, we do not know their precise effects, but we justifiably trust they will be negligible. Such an approach is standard practice in computational chemistry. For instance,

- if we are interested in the heat of formation of a compound, we can avoid the inclusion of entropies;
- if we are not dealing with conical intersections and nonadiabatic effects, we can trust the validity of the Born–Oppenheimer approximation;
- if we do not have reasons to believe in the significant possibility of atomic quantum tunneling, there is no reason to consider the wave function nature of nuclei;
- if we are doing organic chemistry, most probably we will avoid the inclusion of relativistic effects;
- if the HOMO–LUMO gap is considerable, we will not run multireference methods;
- if the solvent is not protic, we may avoid the use of explicit solvent molecules in favor of only applying simple continuum solvent models.

This is an intrinsic part of our job: to sensibly select the computational model that will “truthfully” describe our system, avoiding expensive, nonsignificant computations. As the quote says: “Everything should be made as simple as possible, but not simpler”.^13^

On the other hand, we have the “unknown unknowns”,^14^ all the effects that we are not even aware of, but they shape the reaction in real life, and therefore may severely corrupt any computational result. A famous case was the use of Density Functional Theory (DFT) without dispersion corrections (especially with B3LYP), a standard until the early 21st century; only the developers of functionals were reasonably aware of this critical problem.

There is an active area of research trying to estimate one of the holy grails of computational chemistry, namely the errors of the computed values and of the applied methods.^15–19^ It is easy to obtain such confidence intervals in the wet laboratory (repeat the experiment X times, and calculate the standard deviation), but in the quantum virtual laboratory there are no normal curves to create statistics (except maybe for quantum molecular dynamics, machine learning, and others), and thus these new error analysis techniques are desperately needed. Despite this effort, the fact still stands, we cannot assess the uncertainty of the effects we are not even aware of.

In the condensation reaction of Figure 2, a critical insight provided by quantum chemistry (QChem) was that the proton transfer from the carbon in the acyl anion intermediate to the oxygen in the Breslow intermediate is essentially impossible without a protic cocatalyst (water or alcohol). The determining transition state and the determining intermediate reside in this reaction, and therefore understanding this step is crucial to comprehend the kinetics of the complete mechanism. This secondary proton-shuttle catalytic cycle inside the main one has a strong resemblance to the Grotthuss effect, thus explaining the water’s catalytic nature. This was a complete unknown–unknown, but the necessity of finding a plausible mechanism was imperative. The proposition required the conscious addition of explicit water molecules, a process with not many previous cases reported,^20–24^ and that no automatic or “intelligent” computer agent would consider (unless explicitly trained for this). Therefore, chemical intuition, bibliographical study, and some quantum computational chemistry are necessary to convert the unknown–unknown into a known-(un)­known. It is possible that in this modern AI times this will still be the most important task for experimental quantum chemists.

We can bring another more personal example; in our laboratory we recently embarked on understanding the influence of Quantum Tunneling (QT) in the stability of molecules.^25–31^ Up to a short time ago, the possibility of a strained molecule to decompose by QT was, for us, an unknown–unknown, just an afterthought, an inconsequential effect beyond any logical reasoning, since “quantum tunneling does not occur with heavy atoms”. Evidently we were wrong (at least for specific molecules), since quantum tunneling instability can be a real issue.

Another example of our work involved the mechanism of intramolecular proton transfer. This process required, according to our computations, extreme temperatures, completely at odds with the *in vitro* experiments. After many headaches and hard work we realized that the reaction is impossible without a proton shuttle, usually traces of water in the organic solvent acting similar to the Grotthuss effect depicted in Figure 2.^20,32^ Both the possibility of quantum tunneling instability and of the water bridge cocatalyst mechanism were our long-time unknown unknowns, until we experienced a small eureka moment (interestingly, now we are studying the possibilities of both effects acting together^33^).

The process of uncovering supposedly irrelevant effects is an intrinsic, critical part of every laboratory, but the difference between the wet and the computational labs is clear. In the former, the effect will always be there, even if we are oblivious of it; in the latter, the effect may not be there if we are not explicitly including it in the computational protocol.

### 1.3 Controllable vs Intrinsic Effects

When speaking about known or unknown effects, we can distinguish between two types (Figure 3). Mostly focusing on QChem, but acknowledging that the same analysis can be applied on other branches of computational chemistry, the first group involves the effects that can consciously be included or neglected by adding specific keywords in our favorite software: QT, entropy, multiconfiguration, counterpoise correction, dispersion correction, molecular dynamics, etc.; these are nonstandard effects requiring explicit algorithms that must be activated when needed. The second group are all the effects that, like *in vitro* chemistry, cannot be removed from a well-behaved quantum computation, unless doing some methodological gymnastics: resonance, exchange, polarization, or induction; all of these are intrinsic properties of every quantum method (of course non-quantum methods like molecular mechanics or docking have a different set of controllable and intrinsic properties).

**Figure 3:**
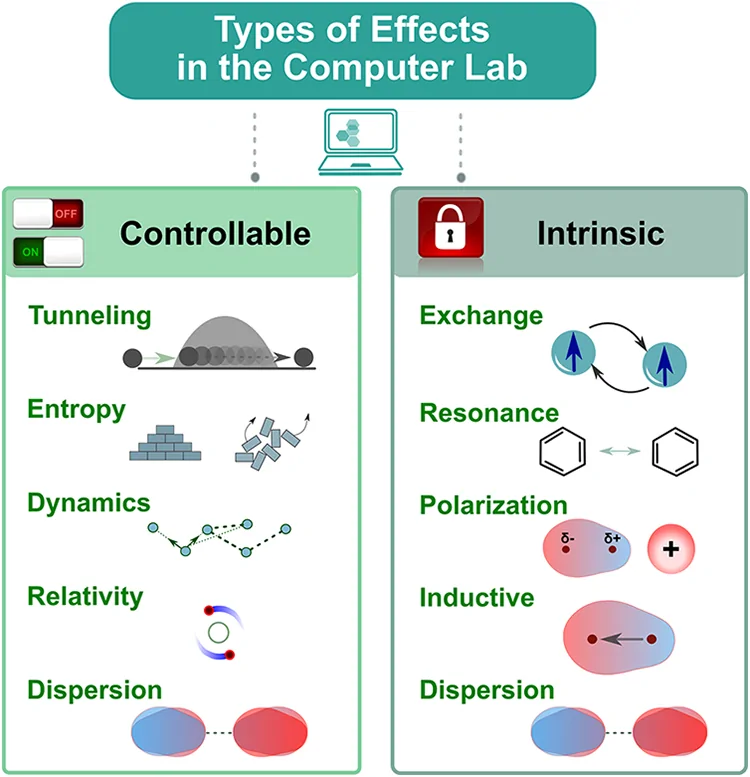
Controllable and intrinsic contributions underlying known and unknown effects in quantum-chemical computations. Note that some effects can be in one or the other column, depending on the theoretical method.

One of the most striking controllable effects is relativity. This is a particularly interesting case, since by its own nature it cannot be part of the QChem body, as it belongs to an orthogonal theory in physics. For heavy atoms, relativity can generate a major change in their chemical properties, especially for the sixth and seventh rows of the periodic table, but with non-negligible effects also in the fifth row. A subtle outcome is that the heavyweight platinum atom is a d^9^s^1^ in its ground state, but its lighter sibling, palladium, is a d^10^, with the concomitant difference in redox chemistry. But more striking effects are the famous facts that gold would not be that stable, golden and shiny,^34,35^ that mercury would melt at ca. 400 K instead of being a liquid at room temperature^36^ (also copernicium, with an atomic number of 112 and a half-life of half a minute, is predicted to be a liquid^37^), or that lead would make a very lousy car battery without relativity^38^ (a celebrated story in the media^39–42^ ).

Relativity is not a standard procedure in quantum computational chemistry. It has to be rationally decided that it is an effect worthy of being considered, and then it must be activated through the proper keywords. This will require deep knowledge of what relativity entails in molecules, as there are many levels and effects applicable to different situations (DKH of different orders, ZORA, X2C, RESC, with or without spin–orbit coupling or quantum electrodynamics corrections,^43,44^ selection of the basis set,^45^ etc.). Again, in the virtual lab it is an effect that must be turned on, but in the real flask it cannot be turned off; a gold ring will always remain golden in the hand of the married.

Noteworthy, there are effects that may be controllable or intrinsic, depending on the method of choice. Possibly the most famous example is the already cited dispersion, aka London forces. For an accurate, “well-behaved” method such as Coupled Cluster [typically CCSD­(T)], it is intrinsic, and cannot be turned off (actually, this is the golden standard to estimate dispersion forces, such as in the S22 and S66 benchmarks).^46^ In contrast, density functional theory methods generally lack this interaction due to the local nature of GGA-based functionals (according to the Hohenberg–Kohn theorems, in the “mythological” perfect functional this effect should naturally be included, but we are not there yet). Notoriously, as said above, for B3LYP there is a complete absence of dispersion forces; this failure is sometimes used as a feature, using it as a simple and fully controllable effect to measure London forces^47^ by turning on and off a dispersion correction.

Relativity certainly is a controllable effect, but can, in practice, be turned into an intrinsic effect by using relativistic effective core potential (ECP) basis sets, since they are almost the default for heavy atoms (such as with the Def2 ^48^ or the PP version of the correlation-consistent^49^ basis set families). This provides the simplest but still very useful approximation, in an almost “relativity for dummies” approach. As with the dispersion correction, switching between basis sets with and without relativistic ECPs can serve as an easy gauge for the impact of this effect.

Another interesting (and also rather obvious) observation is that the controllable corrections can be independent of each other, or they can have an interdependent hierarchy. For instance entropy and relativity are detached add-ons which can individually be turned on, depending on the job. On the other hand, the order of excitations of post-HF methods usually makes no sense without including the previous lower-order terms; there is little sense in computing the third-order perturbational terms of MP3 and not including the second-order ones (there is an exception in configuration of interactions, where the Brillouin theorem makes the use of singly excited Slater determinants of considerably less importance than the doubly excited ones). These added effects and corrections are built as a matryoshka doll, adding layer over layer to improve accuracy or to expand the number of covered described phenomena.

This matryoshka analogy is not intended to represent a strict methodological hierarchy since, as with relativity, many effects can be treated independently depending on the scientific question being addressed (metaphorically Piela compared it to a tree,^50^ where some effects may branch from the main QChem trunk). Rather, it serves as a conceptual framework for organizing progressively richer physical descriptions according to the observables or physical phenomena under investigation.

Along these lines, in this article we will now succinctly but systematically delve into one of these onion-layered series: the gradual inclusion of quantum effects at deeper and deeper levels. We will conceptually describe them, their key observables, how they are modeled, and provide brief, representative examples (both experimental and computational). We will begin at the very beginning, the quantum electron, and we will finish at the very end, the quantum subatomic particles (Figure 4).

**Figure 4:**
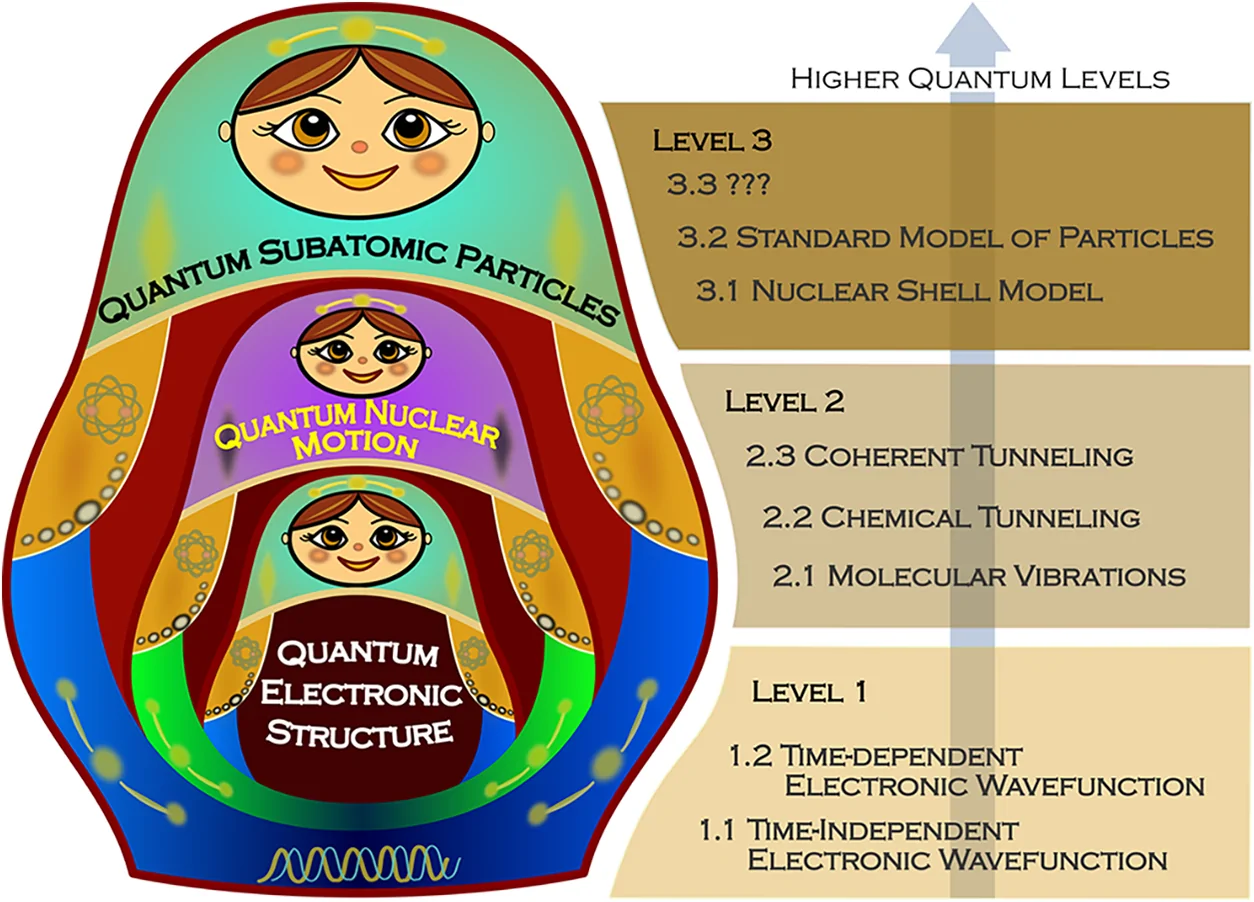
Schematic representation of the hierarchical organization of quantum phenomena using a matryoshka-like analogy.

## 2 Quantum Electrons

### 2.1 Time-Independent Regime

At the deepest and primordial level of the matryoshka, chemistry is governed by electrons as indistinguishable quantum particles without defined paths, only describable by quantum states whose collective properties give rise to molecular stability, reactivity, spectroscopic signatures, or other observables. Within this first level, the distinctions between metallic and insulating materials, as well as the very notion of chemical bonding, arise as direct consequences of quantized electronic states and wave mechanics.

There is computational chemistry without quantum electrons (we may call it “level zero” of the matryoshka), but there is no quantum chemistry without it. QChem can be taken as the study of the electronic wave function, plus small amendments. And this is exactly what we typically do in most laboratories.

As we all know, electrons and nuclei interact with each other in extremely complex and intractable ways. However, it is possible to conceptually isolate the electronic problem for a fixed nuclear framework^51^ with the Born–Oppenheimer approximation (BOA),^52,53^ taking advantage of the much heavier nuclei. As such, the electronic subsystem adapts almost instantaneously to any given nuclear positions, permitting the separation of the heavy and light particles wave functions.^54^ This framework allows the definition of potential energy surfaces (PES) with all that this entails (equilibrium structures, relative energies, vibrations, etc.), and thus provides the natural theoretical basis for this first level of the hierarchy. The BOA is sometimes zoologically likened to flies surrounding elephants, but in truth this is an exaggeration; a proper comparison of the e^–^ and carbon masses indicate that a more accurate analogy would be of a housefly (*Musca domestica*) to a brown rat (*Rattus norvegicus*). Just remember that some flies bite, a metaphorical description of avoided crossings with significant nonadiabatic terms, certain photochemical effects, or some scattering reactions,^55^ among other possible weaknesses of the BOA.

In this first level, therefore, we exploit stationary quantum electronic situations (time-independent) entrapped inside an electrostatic field created by classical nuclei, which define the molecular or crystalline structure. This begs the question of what really is a molecular structure, especially at the light of the BOA, another ontological matter that we leave to philosophers.^56–59^ Nevertheless, this description, if properly applied, already captures the defining features of electronic quantum mechanics, including electron indistinguishability, antisymmetry of the many-electron wave function, exchange interactions, electrostatic interactions, and the Pauli exclusion principle.^60–62^ These purely quantum effects manifest chemically as shell structures, molecular orbitals, steric repulsions, and characteristic bonding patterns.^63^ Taken together, these elements define what is commonly referred to as the electronic structure (and we dare say that it defines chemistry itself).

All this occurs since we naturally consider our electrons to be in a quantum coherent state. Their small masses lead to large de Broglie wave functions with clear quantum behavior for lengths well beyond common molecular dimensions. This permits an easy *coherence*, a polysemic term (that is, a word with multiple related meanings),^64^ but in chemistry it usually means that there is a significant superposition of states, or technically, the system has nonzero off-diagonal elements in its density matrix. This can be exemplified with one of the most important and fundamental set of equations in chemistry and physics (for simplicity written here with real, orthonormal functions):

1 $$\begin{array}{l} \Psi =\left(\phi_{1}+\phi_{2}\right)/\sqrt{2}\\ \rho =\Psi^{2}=\left(\phi_{1}^{2}+\phi_{2}^{2}+2\phi_{1}\phi_{2}\right)/2 \end{array}$$

The typical example of these equations is to take the two ϕ functions as, for instance, the 1s atomic orbitals of two hydrogens, and ψ as the σ bonding molecular orbital of H_2_. The wave function is not a quantum mechanical observable, but its square, the electronic density ρ­(*r*), is, which according to the Born interpretation is the probability to find the system at the considered coordinates. The third term of this density function is the interference, corresponding to the nonzero off-diagonal elements in matricial form, the wave function superposition responsible for the quantum chemical bonding. In QChem, electronic interference is everything, and therefore this is the core of the matryoshka, the first level in the hierarchy of quantum effects. We will return to this basic expression at the end of the article.

#### 2.1.1 “Real-Life” Experimental Observables

As argued above, real-world experiments cannot turn off “undesired” effects, but certain studies can be directed to unveil the pursued ones. In that sense, the electronic stationary wave functional structure cannot be directly accessed, but can be inferred. Several experiments reveal what seems to be orbitals,^65^ and orbitals can be mapped from experiments. However, as philosophers and chemists pointed out, it is hard to argue in favor of the real existence of molecular orbitals when they are strictly non-Quantum Mechanics observables without any specific Hermitian operator (although “existence” may have multiple meanings).^66–72^

What can be readily observed are spatial distribution of the electron density, spectra, or momentum-resolved responses, which encode how electronic states collectively manifest. This may be obtained from diffraction techniques like XRD and electron diffraction.^73–75^ Under appropriate conditions, these measurements yield ensemble-averaged electron density distributions that encode bonding patterns and electronic polarization in molecules and solids.^76^ Energy and momentum-resolved information can be obtained through spectroscopic techniques such as photoelectron spectroscopy, and when combined with inverse photoemission or tunneling spectroscopy, provide access to unoccupied states. Optical absorption yields excitation energies defining an optical gap that reflects, but does not strictly coincide with, the HOMO–LUMO separation. In solids and surfaces, angle-resolved photoemission spectroscopy (ARPES)^77,78^ directly probes electronic band dispersion and constitutes one of the most direct observations at this level. In general, optical absorption and emission techniques^79^ can access electronic energies, while local probes (such as scanning tunneling and atomic force microscopy) can measure the local density of states near the Fermi level and frontier orbitals.^80–82^ These studies actually reflect collective energies and local densities, which after appropriate deconstruction of the signals may be interpreted in terms of molecular orbitals, critically relying on theoretical modeling.^83^ Still, one can argue that even if not strictly quantum observables, they pass the duck test (“if it swims like a duck, and quacks like a duck...”).

#### 2.1.2 Computational Frameworks

This level is the bread and butter of the quantum chemistry community. It encompasses electronic structure methods that solve the time-independent electronic problem under the BOA, yielding stationary electronic properties for fixed nuclear configurations. Even “dynamical” properties, including the estimation of threshold energies, are typically obtained from displacing the atoms, and recomputing the clamp-nuclei electronic structure, plus gradients and second derivatives (Car–Parrinello^84^ and other molecular dynamic methods certainly are more advanced, but start from this level of computations, one direction to make the matryoshka grow that we are not following in this work). Within this level, different methodological frameworks coexist not as descriptions of different physical regimes, but as alternative representations of the same stationary electronic quantum problem.

The “usual suspects” methods are widely known and appear in most QChem books,^54,85–87^ and therefore we only cite them here for the sake of completeness. Wave function-based approaches start by constructing the many-electron wave function by and large from the mean-field approximation of Hartree–Fock (HF).^88,89^ This is improved by the use of perturbational theory on the HF reference (MP2 and further versions), or by judiciously adding excited state Slater determinants in configuration interaction and coupled-cluster approaches, especially with the “golden standard” CCSD­(T). These approaches systematically incorporate electron correlation, mostly of the dynamic type. Static correlation is added by selectively including more optimized references, with the complete-active space (CASSCF) and further developments of this tricky technique. In parallel, DFT^90,91^ reformulates the electronic problem taking instead of the wave function the electron density, embedding many-body effects into the kinetic, electrostatic, and exchange-correlation functionals (with a little help from Kohn–Sham noninteracting orbitals and some HF-type of exact exchange to improve accuracy), enabling an efficient and scalable description of the electronic structure in large molecules, surfaces, and condensed phases. Some modern DFT techniques even started to deal with cases of strong static correlation.^92–94^ These electronic structure approaches apply to finite molecular systems and to extended periodic materials as well. Differences between molecules and solids arise from boundary conditions and basis sets rather than from the underlying electronic theory. Other methods that deserve recognition are Valence Bond Theory^95^ in its several flavors (based on structures close to the chemical intuition), GW approximation,^96^ density matrix renormalization group, quantum Monte Carlo, some composite methods such as G4, W4 and HEAT, etc. And of course, the tentacles of machine learning and similar techniques are currently being introduced^97^ to accelerate the development of electronic structure methods, and quantum computing might overpower classical computing for QChem, always ten years from now.^98,99^

#### 2.1.3 Remarks for the Hierarchy

This first level constitutes the electronic backbone of chemistry and adequately explains molecular and material properties as long as nuclear motion is slow, classical, or effectively averaged. Its limitations become apparent when the electronic density evolves over time. In such situations, what matters is no longer the electronic structure at equilibrium, but its evolution: electronic excitations, relaxation processes, and out-of-equilibrium response cannot be fully captured in a description based solely on stationary eigenstates. At this point, the time-independent framework reaches a conceptual limit, leading us to the next level of hierarchy: time-dependent quantum electrons. Still, whatever extra methodological dimension of quantum is required, it will need to be built over the electronic time-independent regime.

This is a good place to cite another branch of theoretical and computational chemistry, namely statistical mechanics, adequately referred to by a reviewer as “the glue that allows comparison between theory and experiment”. All the results obtained by the ground-state quantum electronic level (plus quantum vibrations, see below) can only be connected to *in vitro* measures by making use of the ergodic hypothesis, the Boltzmann distribution, the partition function, and its subsequent derivations. In the end, the typical information that we obtain in the wet lab is concentrations and rates (except, of course, for the structural information from spectroscopy, microscopy, electrochemistry, and other specific instrumental techniques); thus, statistical mechanics develops the translation tool to decode QChem in the flask.

### 2.2 Time-Dependent Regime

The idea of the matryoshka model is that we judiciously select only the most critical models to computationally tackle the problem at hand, and nothing more. It is a matter of computational and cognitive efficiency. Nevertheless, the basic tenets may not be enough to unlock new effects, and therefore a new layer of quantum techniques must be dressed over the previous one. And most times the next layer of the matryoshka shifts from the stationary electronic structure to explicitly time-dependent electronic behavior. Unlike the previous level, where stationary eigenstates obtained from the time-independent Schrödinger equation approximations provide an adequate description, this level addresses situations in which the electronic system is driven out of equilibrium, either by external perturbation, (such as light, applied fields, or bias) or by the intrinsic redistribution of electron populations following excitations. Therefore, approximations to the time-dependent Schrödinger equation must be sought, where the relevant observables acquire a dynamical character.^100,101^

At this level, electrons can sustain transient superpositions, time-dependent polarization and charge redistribution, and nonequilibrium currents. These phenomena are not limited to molecular photochemistry but also arise in electron-matter interactions, including atomic photoabsorption, scattering-induced excitations, and the dynamical screening response of large, heterogeneous electronic environments. The common physical ingredient is that a description based solely on electronic localization becomes insufficient; instead, one must account for how electronic degrees of freedom respond in time through excitation, screening, and relaxation mechanisms (Figure 5).

**Figure 5:**
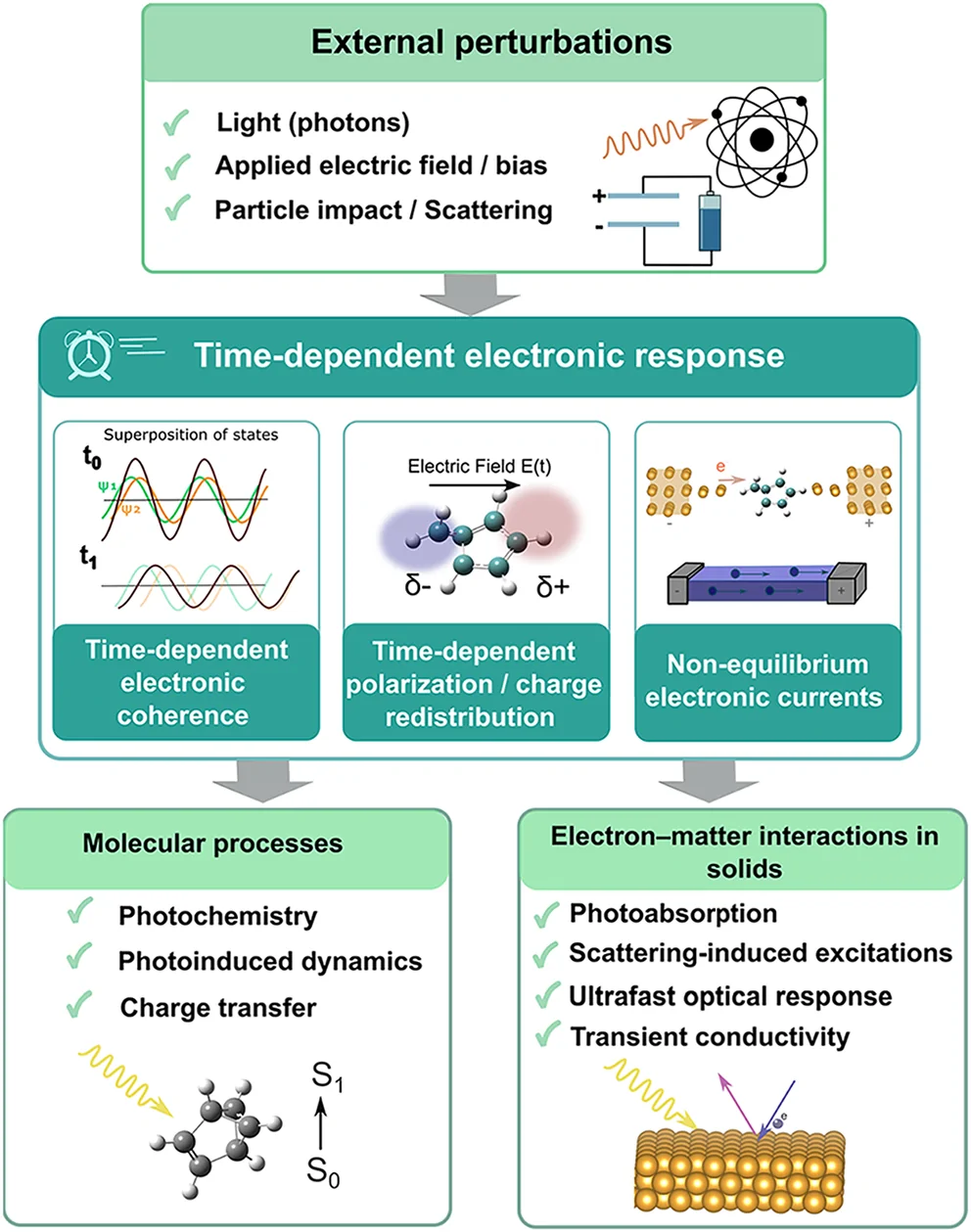
Schematic representation of the processes included in the time-dependent electronic regime. External perturbations, such as light, electric fields, or particle scattering, induce a time-dependent electronic response, leading to transient superpositions, time-dependent polarization, and charge redistribution, as well as nonequilibrium electronic currents. These effects underlie ultrafast molecular processes and interactions between electrons and matter in solids.

Within this regime, electronic modeling becomes intrinsically linked to excitation pathways and experimentally accessible response functions. The BOA still works quite well in many situations, especially when dealing with driven-response problems or solid materials, where the nuclei can be considered fixed or undergoing very slow changes.^102^ But this is where the situation becomes complicated: when multiple electronic states become energetically accessible or near-degenerate, adiabatic descriptions begin to break down, and nonadiabatic physics becomes essential, pushing us toward models that explicitly couple electronic and nuclear dynamics.^103^

#### 2.2.1 “Real-Life” Experimental Observables

At this level experiments no longer probe stationary properties but track how the electronic system evolves following a perturbation, thereby revealing excitation, relaxation, and redistribution processes on ultrafast time scales. The experimentally accessible observables at this level can be broadly classified into population-based, coherence-related, response-based, and emission-related quantities, all of which reflect different aspects of the same underlying time-dependent electronic dynamics. The relevant observables include time-dependent electronic populations, coherences between excited states, charge-transfer dynamics, induced electronic currents and polarizations, as well as ionization yields and energy-resolved photoelectron signals. In this regime, electronic coherence manifests as short-lived superpositions between excited states, giving rise to interference effects in time-resolved spectroscopic signals.^57,64,96,104^

In molecular systems, population-based observables track the evolution and relaxation of discrete excited states, whereas in solids they manifest as nonequilibrium carrier populations, excitons, and transient band occupations. Experimentally, these dynamics are accessed through ultrafast spectroscopies, including pump–probe transient absorption (TA) spectroscopy^105^ and time-resolved photoluminescence (TRPL),^106,107^ which probe excited-state lifetimes and population transfer. Coherence-related observables encode transient electronic superpositions and interference effects, and are accessed through two-dimensional electronic spectroscopy (2DES).^108,109^ Response-based observables, such as transient photoconductivity and carrier mobility, are probed by time-resolved terahertz spectroscopy (TRTS),^110,111^ while electron energy-loss spectroscopy (EELS) probes dynamical electronic screening and collective excitations.

Finally, emission- and energy-resolved observables provide direct access to electrons escaping the system under nonequilibrium conditions. Time-resolved photoelectron spectroscopy (TRPES)^112,113^ and time- and angle-resolved photoemission (tr-ARPES)^114,115^ map the evolution of electronic energies, band populations, and kinetic-energy distributions of emitted electrons, while ionization yields quantify time-dependent electron emission probabilities. Time-resolved X-ray spectroscopies (tr-XAS, tr-XPS)^116,117^ further provide element-specific insight into transient local electronic structure, linking ultrafast electronic dynamics to transient electronic structure and collective response phenomena.^118^

#### 2.2.2 Computational Frameworks

From a computational perspective, this level encompasses theoretical approaches that describe electronic excitations and explicitly time-dependent electronic behavior. It moves beyond stationary ground-state electronic structure, but it starts from it. Different formalisms (density-based, wave function-based, and Green’s-function approaches) do not correspond to different physical regimes, but rather to alternative representations of the same underlying time-dependent electronic problem, each tailored to access specific observables with different accuracy and computational costs.

A first and widely used route focuses on electronically excited states and linear-response properties, where time dependence is typically accessed in the frequency domain. The workhorse^119^ of these computations is Time-Dependent DFT (TD-DFT),^102,120–124^ which provides an efficient framework to compute excitation energies, oscillator strengths, and response functions in large systems. Wave function-based excited-state methods, such as equation-of-motion coupled cluster (EOM-CC)^125,126^ and algebraic diagrammatic construction schemes, offer systematically improvable descriptions when higher accuracy (with higher cost!) is required. In extended and periodic systems, electronic and optical excitations are often treated within many-body perturbative frameworks, most notably through the combination of the GW^96,127^ approximation and the Bethe–Salpeter equation.

When the phenomenon of interest is intrinsically driven in time, a complementary strategy consists in explicitly propagating the electronic degrees of freedom in the time domain. Real-time formulations, such as real-time TD-DFT,^128,129^ allow the simulation of ultrafast polarization dynamics, induced currents, and nonequilibrium electronic response under external perturbations, providing direct access to time-resolved observables beyond the linear-response regime.

At the edge of this level, electronic relaxation and branching pathways increasingly reflect coupling with nuclear motion. In situations where nuclear dynamics mediate transitions between electronic states, mixed quantum–classical and nonadiabatic approaches, such as Ehrenfest dynamics and surface-hopping schemes, become necessary.^130^ These methods go beyond the single-surface Born–Oppenheimer picture by allowing transitions between electronic PES through nonadiabatic couplings, while the nuclei continue to propagate as classical trajectories. Such situations commonly arise near avoided crossings and conical intersections, where the adiabatic separation between electronic states breaks down and nonadiabatic couplings dominate the dynamics.

#### 2.2.3 Remarks for the Hierarchy

Although the current level focuses on electronic dynamics, the quantities that can be observed experimentally in real systems often exhibit a vibrational structure derived from electron–nucleus coupling. In a purely time-dependent electronic description, nuclear motion is normally treated as a classical coordinate or as a weak perturbation of the electronic degrees of freedom.

This approximation is insufficient when nuclear motion itself must be described from the point of view of quantum mechanics. The characteristics of this regime include zero-point energy effects, strong isotopic dependence, and tunneling phenomena. In such situations, nuclei no longer follow well-defined classical trajectories but occupy quantized vibrational states that actively influence the dynamics. This marks the transition to the next level of the hierarchy, where nuclear quantum effects become essential.

## 3 Quantum Nuclei

### 3.1 Vibrations and Zero-Point Energy

Following our matryoshka analogy, the next layer of description emerges when the quantum nature of nuclei is explicitly considered. It is commonly said that we should treat nuclei as classical, point-like particles; but this is clearly wrong, since quantized nuclei is part of the standard battery of quantum computations we usually perform, at least in a relatively simple approach.

While the previous levels focused primarily on electronic degrees of freedom, many molecular properties cannot be fully understood from an electronic description alone. Equilibrium structures, conformational stability, and relevant aspects of chemical reactivity are often influenced by nuclear quantum effects (NQEs),^131,132^ requiring an additional level of description beyond the quantum electronic description alone (note that NQEs presupposes quantum electrons in their ground state, thus being an upper layer in the matryoshka model, but not necessarily time-dependent electrons).

Within the BOA, the nuclei move on the PES determined by the electronic structure and their own Coulombic forces. However, the nuclei occupy quantized vibrational states rather than following classical trajectories. This means that their movement is not continuous through reactions and vibrations as classical particles would do, like in the perfectly smooth Hooke’s spring law. On the contrary, even “heavy” particles can only occupy specific energies and positions, and evidently the lighter the atoms the larger the distance between states. This quantization is important in the minima of the PES, especially for high vibrational frequencies, but due to anharmonicities it gets diluted closer to transition states.^133^ We naturally consider this level of quantized nuclei when computing molecular frequencies, a standard practice. But even if we are uninterested in analyzing the vibrations of the normal modes and the IR and Raman spectra, we still need this to obtain the zero-point energy (ZPE) of the molecules and to understand the system’s thermal contributions to produce correct energy values. This means that to obtain the PES indeed we use classical nuclei, but on that we must dress a first layer of NQEs.

Solving the nuclear Schrödinger equation on the electronic potential energy surface yields the quantized vibrational eigenstates that describe the nuclear wave function of the system.^87,134^ But in practice we mostly work within the much simpler harmonic approximation, whose vibrational energy levels in a stable state are given by

2 $$\begin{array}{l} E_{n}=\left(n+\frac{1}{2}\right)h\nu \\ \nu =2\pi \sqrt{k/\mu } \end{array}$$

where *n* = 0,1,2... denotes the vibrational quantum number, ν is the vibrational frequency, μ is the reduced mass, and *k* is the oscillator constant, that is the strength of the bond.^87^ If necessary, anharmonic corrections of simple factors^135,136^ can be included to improve these equations. Most importantly, with *n* = 0, the resulting *hν*/2 term corresponds to the ZPE, indicating that nuclei retain finite motion even in the lowest-energy state. This residual motion is an intrinsic consequence of quantum mechanics and reflects the impossibility of simultaneously localizing a particle with zero momentum, as implied by the Heisenberg uncertainty principle, arguably one of the most “quantum” concepts in physics, here applied to “heavy” particles.

As a result, NQEs influence a wide range of molecular properties. Differences in ZPE between isotopes (especially H/D), for example, can modify reaction energetics and give rise to measurable kinetic isotope effects (KIEs),^137,138^ reflecting the influence of nuclear mass and vibrational quantization on chemical reactivity. It also permits the estimation of the partition function to calculate enthalpies and Gibbs energies, and of the vibrational spectra. The NQE can be naturally extended to periodic solids, especially their phonons (the lattice collective quantized normal mode vibrations), including the crystal ZPE.^139^ These quantum characteristics of nuclear motion are not mere theoretical constructs but give rise to experimentally accessible observables (Figure 6).

**Figure 6:**
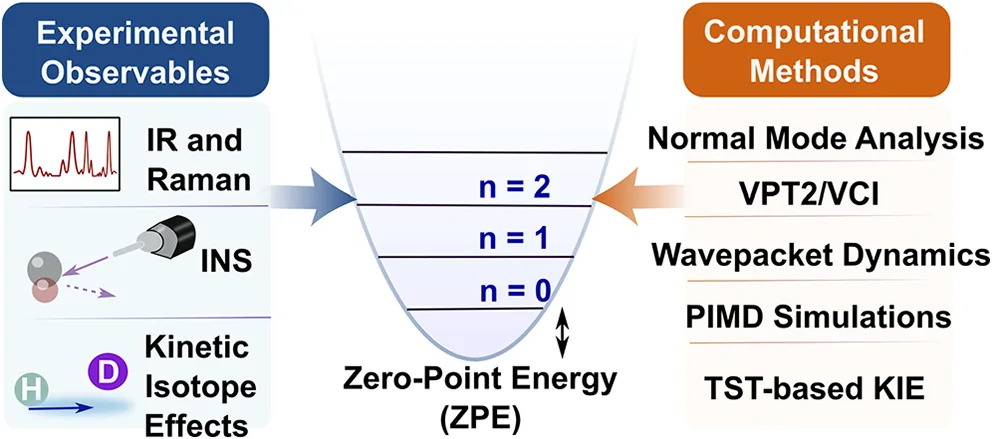
Basic quantum nuclear effects, with nuclei occupy discrete vibrational states on the electronic potential energy surface.

#### 3.1.1 “Real-Life” Experimental Observables

The quantum nature of nuclear motion becomes experimentally accessible through a variety of spectroscopic and kinetic observables. Although the ZPE itself is not a directly measurable quantity, its presence can be inferred from spectroscopic measurements and isotope-dependent effects.

The most direct evidence comes from vibrational spectroscopy, where transitions between quantized vibrational levels appear as discrete spectral features in infrared and Raman spectra.^134,140^ In IR spectroscopy, vibrational transitions are observed when they involve a change in molecular dipole moment, while Raman spectroscopy probes vibrational modes associated with changes in molecular polarizability. These spectroscopic measurements probe the vibrational energy spacing predicted by quantum mechanics.

Inelastic neutron scattering (INS)^141^ offers another powerful tool for studying nuclear dynamics. Since neutrons interact directly with atomic nuclei rather than with the electron cloud, this technique provides particularly direct access to vibrational excitations and lattice dynamics in both molecular and condensed phases such as liquids and solids.

Another important manifestation of nuclear quantum effects arises from isotopic substitution. Since vibrational frequencies depend on nuclear masses (μ in eq 2), replacing atoms with heavier isotopes leads to measurable shifts in vibrational spectra and changes in reaction energetics. These isotope-dependent effects can be observed experimentally through vibrational spectroscopy and through KIE measured in chemical reaction rates.^137,138^

These signatures demonstrate that nuclear motion in molecular systems and solids is inherently quantum mechanical and motivate the development of theoretical and computational approaches capable of describing NQEs.

#### 3.1.2 Computational Frameworks

From a computational point of view, NQEs are most commonly described within the harmonic approximation, where vibrational frequencies, infrared and Raman intensities, and ZPE are obtained through normal-mode analysis. In this framework, the Hessian matrix of the second derivatives of energy with respect to nuclear coordinates is diagonalized to obtain the normal modes of vibration and their corresponding frequencies.^140^ As said above, this is standard procedure in most molecular quantum computational projects.

To consider deviations from harmonic behavior, anharmonic vibrational methods have been developed. Among the most widely used approaches are vibrational perturbation theory (VPT2),^142^ which treats anharmonic effects perturbatively, and vibrational configuration interaction (VCI),^143^ which provides a variational treatment that captures vibrational correlation and mode coupling more explicitly.

Beyond vibrational treatments around a potential minimum, NQEs can also be incorporated into molecular simulations using quantum molecular dynamics approaches. Wave packet dynamics allows for explicit quantum propagation of nuclear wave functions and is mainly applied to small molecular systems.^144^ In contrast, path integral molecular dynamics (PIMD)^132^ provides a statistical mechanical framework for incorporating nuclear quantum delocalization in finite-temperature simulations of condensed-phase systems.

These theoretical approaches allow us to predict isotope-dependent effects and reaction kinetics. According to the conventional transition state theory (TST),^145^ chemical reactions occur through thermal activation over the potential energy barrier, and differences in vibrational frequencies and ZPEs between isotopologues give rise to kinetic isotope effects.^138,146^ This is because in the TS the normal mode of the reaction is imaginary, and therefore without ZPE, thus the ZPE difference in the reactant significantly affects the barrier height (this is for a primary KIE; for secondary KIE the effects are much smaller). A well-known example is the cleavage of C–H bonds versus C–D bonds in organic reactions, where the higher ZPE associated with the C–H stretching mode effectively lowers the activation barrier and results in faster reaction rates for the lighter isotope (∼7 times faster at room temperature).^146^

This KIE effect has been exploited in new drugs and, curiously, in the development of deuterated alcoholic beverages aimed at reducing hangover symptoms.^147^ Hangover is, technically, not caused by ethanol, but by acetaldehyde, the oxidation product of alcohol dehydrogenase. If posterior oxidation with aldehyde dehydrogenase does not occur fast enough, the aldehyde has time to wreak havoc in the body. Deuterium substitution slows the alcohol dehydrogenase oxidation of ethanol through a primary KIE, thereby reducing acetaldehyde accumulation, and avoids the hangover symptoms that many of us are familiar with. This research, centered on NQEs, has proven to deliver successful tales for academic social gatherings.

At sufficiently low temperatures the KIE should exponentially grow according to an Arrhenius trend. However, the semiclassical thermal reaction will probably make the rates too slow to study, and only nuclear tunneling can contribute significantly to it. But we are getting ahead of ourselves, as this effect is not included at this level of the matryoshka!

Computational treatments of the nuclear quantum effects causing zero-point energy contributions are therefore essential for interpreting a variety of experimentally observed phenomena, including reactivity changes arising from isotopic substitution and differences in conformational stability. Moreover, they are easily done in most quantum chemistry software.

Beyond these energetic and kinetic observables, NQEs can also manifest in the electronic density itself. Approaches such as nuclear–electronic orbital density functional theory (NEO–DFT), which treat selected nuclei quantum mechanically alongside the electrons, provide direct access to this complementary perspective.^148^ This points to a broader view of nuclear delocalization, which becomes central in the next level when tunneling is considered.

#### 3.1.3 Remarks for the Hierarchy

Within our matryoshka model, this level describes the most basic, but ubiquitous, quantized nuclear motion. In this regime, nuclear quantum effects manifest mainly through vibrational energy levels, ZPE, and isotope-dependent phenomena, such as the semiclassical KIE. Of note, in this article we use the term “semiclassical” to indicate nontunneling rates, but including nuclear quantum vibrations (as defined in Bell’s book^149^). There are QT methods that do not strictly use wave functions to calculate rates (like SCT and instanton),^150^ thus also being called semiclassical. This fact generates a very confusing jargon in the field. We will explicitly identify these methods as “semiclassical-tunneling”, hoping to avoid a minor quantum Tower of Babel.

The description provided at this level assumes that nuclear motion remains confined to small oscillations within a single potential well on the potential energy surface, or at the most long-range effects for state functions that do not depend on the trajectory (such as the threshold or the reaction energies). While ZPE and KIE can be accurately treated within this framework, longer trajectory processes fall outside its scope. In particular, the tunneling effects require methods from another layer, in which the nuclear motion is no longer limited to small oscillations but can traverse potential barriers.

### 3.2 Localized (“Chemical”) Quantum Nuclei Tunneling

The new layer of our matryoshka expands the description of nuclear quantum effects beyond localized vibrational motion to encompass barrier-crossing processes enabled by QT. Unlike the previous level, in which nuclei are effectively confined to small-amplitude oscillations within a single potential well, this level includes situations in which nuclear motion is no longer limited to a single minimum, but acquires a finite probability of accessing neighboring regions of the PES through classically forbidden pathways.

At this level, the classical picture of an impenetrable barrier separating adjacent minima no longer holds, since even in the absence of sufficient thermal energy (i.e., at cryogenic temperatures), nuclei can cross the barrier and induce structural rearrangements, configurational transformations, or bond-breaking processes.^149^ Note that this section deals exclusively with atomic and molecular tunneling. Electrons can evidently tunnel, but except for studies on electrical conductivity, and considering their much faster QT due to their low mass and much larger de Broglie wavelength, we mostly take this process for granted, and take the electrons as instantaneously delocalized throughout the molecule.

Typically, two regimes are considered: extremely low cryogenic conditions, with the QT process occurring entirely from the ground vibrational state (sometimes called “deep tunneling”), and moderately high temperatures, where the QT process occurs mostly from higher vibrational states, where the barrier is lower and shorter (thermally or vibrationally activated tunneling regime), as shown in Figure 7. At high enough temperatures the classical thermal reaction overpowers the QT; but what means “moderate” or “high” temperatures is strongly systems dependent.

**Figure 7:**
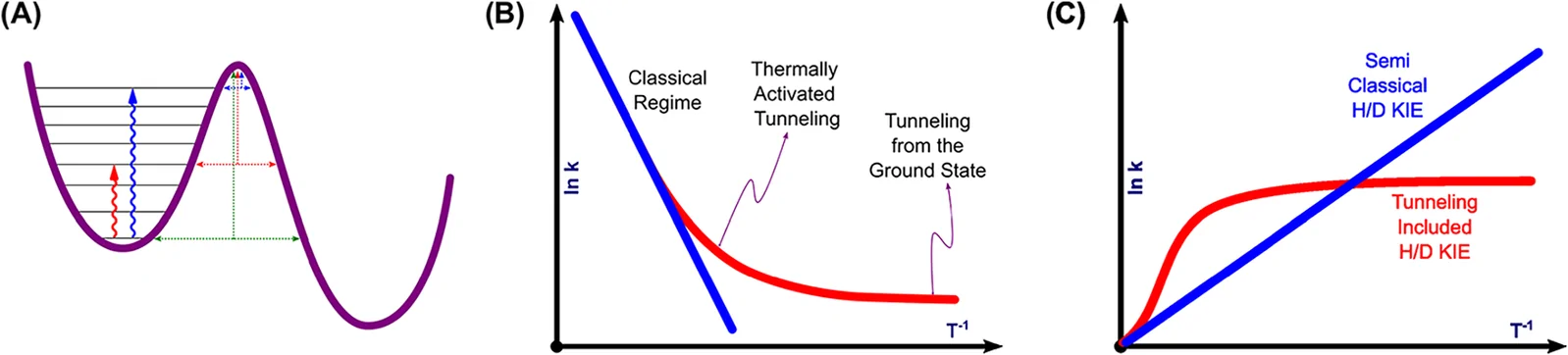
(A) Quantum tunneling from different vibrational states, showing the lower and narrower barrier at higher states. (B) At deep cryogenic conditions QT may occur exclusively from the ground state, making it independent of the temperature. At moderate *T*, according to Boltzmann distribution, some vibrational states can be populated, making the QT reaction more probable from them; the tunneling itself is not temperature-dependent, but the occupation of higher vibrational states is. At high *T*, the process is essentially equivalent to the transition state theory. (C) Typical H/D KIE profile, starting from a fully thermal regime at high *T*, QT mostly for hydrogen in the middle, and fully deep tunneling for both H and D at low *T*. Adapted with permission from ref 151. Copyright 2020 Royal Society of Chemistry.

In the thermally activated regime we usually speak about the tunneling transmission coefficient, κ, a factor of the rate constant that estimates the QT. Based on TST, and neglecting other effects,

3 $$k=\kappa \frac{k_{B}T}{h}e^{-\Delta G^{‡}/\mathrm{RT}}$$

with κ = 1 meaning no tunneling, and κ = 2 setting *T*_C_, the crossover temperature where thermal and classical rates are equal, and below which QT takes the advantage (classical reactivity is more sensitive to the temperature than QT).^149^ This formulation serves as a bridge between classical kinetics and quantum nuclear dynamics. Interestingly, in rare cases κ can be smaller than one, making the reaction slower than the semiclassical mechanism; the wave function nature of the atoms can produce a chemical reaction when there is no energy for that, but it might also hinder it (“rebound”) when there is.

For deep QT, speaking in terms of κ makes little sense, as it will take humongous values. At this low temperature regime, the ground state QT is the only significant term. In this case, we have pure reactivity due to nuclear wave function delocalization. A rough approximation of the reaction rate constant can be obtained directly from the transmission probability calculation of an impacting quantum particle on a model barrier of an arbitrary shape (rectangular, parabolic, etc.). That is, what will be the square of the forward-moving nuclear wave function at the other side of the barrier, compared to the rebound, backward-moving wave function without crossing the barrier. In a chemical reaction, the number of impacts will depend on the vibrational normal mode leading to the reaction coordinate, with the frequency of impacts (ν) multiplied by the probability of QT (*P*):^149,151^

$$\begin{array}{c} k=\nu_{R}P\\ P=e^{-2\theta }\\ \theta \approx \mathrm{xw}\sqrt{2\mu \Delta E^{‡}}/\hbar \end{array}$$ 4

where θ is the action, *w* represents the effective width of the barrier (i.e., the spatial extent of the classically forbidden region), *x* is a factor dependent on the shape of the barrier, μ is the reduced mass associated with the reaction coordinate, Δ*E* ^‡^ corresponds to the threshold energy, and *ℏ* to the reduced Planck constant.^25^ This expression highlights that tunneling depends not only on the barrier height, but also on the geometric characteristics of the PES. In particular, the width of the barrier proves to be a decisive factor: even tall barriers can be traversed if they are sufficiently narrow, while wide barriers can effectively suppress the tunneling effects even when their height is modest.^152,153^

This exponential dependence on the barrier width also provides a natural explanation for a seemingly counterintuitive and infrequent, but certainly possible, aspect: the ability to undergo QT with heavy atoms (that is, more massive than hydrogen). Although an increase in mass significantly reduces the probability of QT, this effect can be compensated for when the barrier is sufficiently narrow, or the shape of the potential energy surface is favorable. Consequently, the QT effect is not limited to light particles, but can also play a significant role in rearrangements involving carbon, oxygen, and even fluorine.^154–158^

From a conceptual standpoint, tunneling enables barrier-crossing transformations between well-defined molecular configurations. This framework encompasses asymmetric double-well potentials, degenerate double-well systems described in a localized representation, fluxional molecules characterized by multiple equivalent minima on the PES,^159^ and metastable structures that evolve toward more stable configurations along exothermic pathways. Consequently, at this quantum level, the existence of a local minimum on the PES is no longer sufficient to guarantee kinetic stability, since tunneling introduces finite lifetimes even at very low temperatures.

It is important to note that the tunneling processes analyzed at this level can typically be described within a single PES under the BOA,^132,160^ preserving the separation between electronic and nuclear motion. At this level, nuclear quantum effects extend beyond localized vibrational motion to enable barrier-crossing dynamics between well-defined minima. However, in systems where barrier crossing is accompanied by significant electronic reorganization or strong coupling between electronic and nuclear degrees of freedom, the separation between nuclear and electronic motion may become less clear, and multidimensional descriptions of the reaction coordinate may still be required.^132,160,161^

#### 3.2.1 “Real-Life” Experimental Observables

Experimentally, QT is not a direct observable, but is inferred from characteristic deviations from classical behavior. Likewise, the question of how long a system spends traversing the classically forbidden region does not possess a uniquely defined observable answer.^162^ In quantum mechanics, time enters as a parameter in the time-dependent Schrödinger equation rather than as a Hermitian operator, and therefore no unique operator can be associated with a tunneling duration.^162^ Consequently, tunneling time cannot be interpreted as a classical transit time across the barrier, but rather as a characteristic time scale associated with the quantum transition between states localized on opposite sides of the barrier, defined operationally through dynamical models that relate measurable observables to the underlying quantum dynamics.^162,163^ In the deep tunneling regime, theoretical analyses predict only a weak dependence of this effective duration on barrier width (Hartman effect), further illustrating the nonclassical nature of barrier penetration and reinforcing the interpretation of tunneling time as a derived rather than directly measurable quantity.^127,128^

Consequently, experimental identification of tunneling relies on indirect signatures that reveal departures from classical over-the-barrier dynamics. One of the most prominent signs is the temperature dependence of reaction rates (Figure 7B). In semiclassical, thermal reactions, and according to Arrhenius empirical studies, Eyring’s transition state theory, and in general according to statistical mechanic principles based on the Boltzmann distribution of excited vibrational states, the reaction rate constant diminishes with the temperature, in principle with a linear trend between log *k* vs *T* ^–1^, with an activation energy and preactivation constant of

$$\begin{array}{c} E_{a}\equiv -R\frac{\partial \ln k}{\partial T^{-1}}\\ A\equiv k\left(T=\infty \right) \end{array}$$ 5

However, in regimes dominated by the tunnel effect the classical Arrhenius behavior ceases to hold, often resulting in a weak dependence of *k* on the temperature.^164^ Therefore, if the Arrhenius graph turns into a “subArrhenius regime”, with lower than expected *E*_a_ and *A*, then there is high probability of significant thermally activated QT.^165^ And of course, if the rate constant is practically independent of the temperature, with no appreciable threshold energy, then there is deep tunneling. It is worth mentioning that *all* chemical reactions will produce a subArrhenius graph like the one of Figure 7B, since sooner or later, at low enough temperature, the QT mechanism must overcome the semiclassical, thermal one; however, if the QT lifetime is longer than the age of the universe, then we can safely discard this layer of the matryoshka.^166^

KIEs values are a particularly sensitive indicator of the QTE. While semiclassical KIEs are primarily due to differences in ZPEs, the tunnel effect introduces an additional exponential sensitivity to the nuclear mass. As a result, isotopic substitutions can produce changes in reaction rate that significantly exceed classical predictions, leading to unusually large KIEs, a clear signature of tunnel contributions.^137,167^ Note that at very low temperatures, both isotopes will be at the deep QT regime, making the KIE profile independent of the temperature (and lower than the extrapolated KIE for the thermal reaction), as shown in Figure 7C. Thus, a KIE independent of the temperature is a sign of deep tunneling, as can also appear in an abnormal Swain-Schaad relationship (the ratio between H/D/T rates).^168^

Another obvious key experimental signature is the persistence of reactivity at cryogenic temperatures, where classical over-the-barrier activation becomes negligible. The observation of finite reaction rates under such conditions, for instance by IR,^169^ provides compelling evidence of quantum barrier penetration and, in many cases, indicates tunneling-driven decay or rearrangement of metastable species.^170,171^ Of note, certain reactions can change their selectivity and generate two different products at different temperatures if one of the barriers is low and broad, favoring the thermal reactivity, and the other is high and narrow, favoring QT; this is what was called tunneling control.^170,172^ In extreme cases, this can create a selectivity divergence due to strong QT KIE, in what was called isotope-controlled selectivity.^173,174^

Taken together, these signatures—non-Arrhenius temperature dependence, enhanced isotope effects, and reactivity at low temperatures— define the experimental fingerprint of tunneling-driven processes, providing a direct benchmark for theoretical descriptions and linking measurable kinetics to the rate constants predicted by quantum-corrected TST.

#### 3.2.2 Computational Frameworks

Within a one-dimensional semiclassical-tunneling approximation, barrier-crossing events can be approximated in terms of transmission through model potential energy barriers of different shapes (e.g., rectangular, parabolic, or bell-shaped potentials) which have analytical solutions (similar to eq 4), or through more accurate numerical integration schemes such as the Wentzel–Kramers–Brillouin (WKB) formalism with arbitrary shaped barriers.^149^ Even simpler, for thermally activated QT the Wigner (κ_W_) or the slightly improved Bell (κ_B_) formulations^149^ introduce a low-order correction to the classical rate constant based on the local curvature of the potential energy surface at the transition state,

6 $$\begin{array}{l} \kappa_{W}=1+\frac{1}{24}{\left(\frac{h\nu_{‡}}{k_{B}T}\right)}^{2}\\ \kappa_{B}=\frac{h\nu_{‡}/2k_{B}T}{\sin\left(h\nu_{‡}/2k_{B}T\right)} \end{array}$$

with ν_‡_ being the imaginary frequency; this is a ballpark estimation near the crossover temperature.^149^ From this equation, it can be derived that when *T*_C_ is set to room temperature, ν_‡_ ≳ 1000 cm^–1^, making chemical QT almost exclusively possible with H transfer reactions.^175^

However, if accuracy is sought, then there is a plethora of effects that must be switched on. While conventional TST defines the dividing surface at the saddle point (i.e., the transition state separating reactants and products), variational transition state theory (VTST) relaxes this constraint by allowing the location of the dividing surface to vary along the reaction coordinate. In this framework, the rate constant is obtained by minimizing the reactive flux, thereby reducing recrossing effects and providing a more accurate classical reference for the inclusion of tunneling contributions.^176,177^

Within the VTST framework, tunneling is often incorporated through semiclassical QT multidimensional corrections that account for deviations from the minimum energy path (MEP). In realistic molecular systems, tunneling is inherently multidimensional, involving collective nuclear motion and coupling between the reaction coordinate and transverse vibrational modes. This would minimize the action (eq 4) by modifying the effective barrier shape, reducing the barrier width even if it is at the cost of a higher barrier, thus maximizing the rate.^178^ An example of one of these methods is the small-curvature tunneling (SCT), which describes the tunneling near the MEP, and incorporates the local curvature of the potential energy surface, evaluated using the second derivatives along the reaction coordinate; to this, it adds the possible vibrational contributions associated with thermally populated excited states.^179^ In contrast, large-curvature tunneling (LCT) considers trajectories that strongly deviate from the MEP and drastically “cut corners” in the configuration space.^179^ Microcanonical optimized multidimensional tunneling (μOMT)^180^ combine SCT and LCT simply by selecting the dominant tunneling method at a given energy. It is important to point out that these semiclassical tunneling methods, even without explicitly considering the wave function nature of the nuclei, still capture the main outcome of their quantum behavior. They bring an excellent compromise between accuracy and computational cost, making them one of the only realistic options for the extremely time-consuming estimations of QT rates in small and medium size molecular systems.

Beyond semiclassical-tunneling treatments, more rigorous quantum approaches such as instanton theory describe tunneling as a periodic trajectory in imaginary time, offering an accurate description in the deep tunneling regime.^160,181^ In parallel, PIMD provides a framework for incorporating NQEs into dynamical simulations, enabling the treatment of tunneling in complex and condensed-phase environments.^132^

In practice, reliable predictions of tunneling rates require not only an accurate treatment of nuclear quantum effects, but also, and maybe more important, a high-quality PES. The interplay between barrier height, barrier width, and multidimensional coupling ultimately determines the tunneling probability, highlighting the importance of an accurate description of PES for reliable kinetic predictions. This implies that, unless a proper benchmark is performed, DFT cannot approach accuracy, and much more involved electronic structure methods are required (that is CCSD­(T) for single reference barriers, or high level multireference methods otherwise).^182,183^

Many recent studies, including contributions from our group, have applied these theoretical frameworks to a variety of molecular systems, demonstrating that heavy-atom tunneling can govern both structural rearrangements and kinetic stability across diverse chemical environments.

#### 3.2.3 Representative Systems

Heavy-atom tunneling governs the dynamics in various chemical systems, including organic rearrangements and conformational transitions, demonstrating that even if hydrogen transfers provide the lion’s share of QT, it is definitely not limited to it. Systems characterized by low and especially narrow barriers provide paradigmatic examples, for example antiaromatic molecules undergoing carbon-tunneling-driven π-bond rearrangements enabled by minimal geometric distortion.^184–188^

While deep QT is independent of the temperature, thermally activated QT is not. At high temperatures, the Boltzmann distribution dependence of the latter makes it converge to the nontunneling formulations like TST (Figure 7B). As a result, it is extremely uncommon to find reactions with significant QT when “heavy” (i.e., non-hydrogen) atoms are involved at room temperature. Even for H-transfers, the transmission coefficient or the KIE will reach three-digit numbers only in very unique cases,^33,175^ and they are heavily influenced by the environment. High temperature QT must be considered, but only if accurate computations are pursued.^165,189^ However, sometimes there are unexpected surprises.^184–187,190,191^

Deep tunneling is another story. The non-QT dependence on the temperature is much more pronounced, and therefore at cryogenic conditions if there is a chemical reaction, it will probably be in the QT domain. It is here where the most interesting effects and reactions can be detected, from heavy-atom QT,^157,184,192–194^ tunneling controlled reactions,^170,172,195,196^ isotope controlled reactions,^173,174^ QT instability,^26–28,31^ domino tunneling,^197^ QT ring opening,^198–201^ nonadiabatic QT,^161^ etc. It is in this low temperature regime where if something happens, this level of the matryoshka must be unlocked. However, care must be taken since sometimes external influences might mirror QT behaviors,^169^ providing false QT positives.^202^

#### 3.2.4 Remarks for the Hierarchy

Level 2.2 describes tunneling between localized nuclear configurations, where its presence is inferred from kinetic observables such as rate constants and KIEs, that is purely chemical effects. Chemical tunneling might be the most critical “unknown–unknown” of this hierarchy, since it is an effect very rarely considered in computational works. Granted, nuclear QT is not common and therefore neglecting it will hardly affect the accuracy of most quantum computations; still, in some cases this has brought critical mistakes that hindered a correct description of reality.

When two minima become (quasi) degenerate and the QT effect between them is substantial, the double-well potential can sustain delocalized eigenstates spanning both wells. This change reflects a shift in regime rather than in mechanism: tunneling might no longer be expressed as population transfer between localized states, but as a quantum superposition, generating an energy splitting between symmetric and antisymmetric eigenstates, marking the transition to the next level.

### 3.3 Coherent (“Physical”) Quantum Nuclei Tunneling

This level focuses on observables arising from delocalized nuclear eigenstates, rather than describing tunneling in terms of reaction pathways connecting localized configurations. Within the matryoshka framework, this level corresponds to a distinct type of experimental observables associated with nuclear quantum coherence, as well as a different type of computations.^183^ Unlike the previous level, in which QT effects manifest through kinetic observables such as rate constants, this regime is characterized by spectroscopically resolvable energy splittings (Δ*E*_01_) between stationary nuclear states, which can be measured using high-resolution spectroscopic techniques.

At this level, we focus on symmetric nuclear systems described by a double-well potential energy surface, which may support nuclear quantum tunneling at very low-temperature. Such potentials typically originate from two symmetry-related molecular configurations with equivalent minima on the potential energy surface, which may arise from inversion, proton transfer, conformational change, or bond rearrangements, all consequences of one of the many Jahn–Teller effects.^203^ In a localized picture (the focus of the previous level), the system can be represented as occupying one of the two minima, which we can call left and right states (**|** ***L*** **⟩** and **|** ***R*** **⟩**). Strictly speaking, these are not stationary solutions of the nuclear Hamiltonian, since the quantum mechanical coupling between the wells mixes both configurations, leading to eigenstates that are spatially delocalized across the barrier region. This is akin to the σ and σ* orbitals in H_2_, produced by the superposition of two s atomic orbitals, except that here the superposition is defined along nuclear rather than electronic coordinates.

This situation is conceptually analogous to the well-known *gedankenexperiment* of Schrödinger’s cat, which illustrates the quantum superposition of classically distinct alternatives. In contrast with the hypothetical superposition of the breathing and the deceased feline (a “zombi” state, as it was once called), the molecular wave function may exist as a coherent superposition of the |*L*⟩ and |*R*⟩ configurations by preserving phase coherence to give rise to experimentally measurable tunneling splittings:

7 $$\begin{array}{l} \left|\psi_{0}\right\rangle =\left(\left|L\right\rangle +\left|R\right\rangle \right)/\sqrt{2}\\ \left|\psi_{1}\right\rangle =\left(\left|L\right\rangle -\left|R\right\rangle \right)/\sqrt{2}\\ \Delta E_{01}=E\left(\psi_{1}\right)-E\left(\psi_{0}\right) \end{array}$$

which is the key observable of this matryoshka level (compare this with eq 1 for electrons). Thus, in this regime tunneling manifests as a spectroscopic signature of nuclear delocalization rather than a chemical transformation. The most classic example is the ammonia umbrella inversion, in which the molecule tunnels through a planar transition state, giving rise to a tunneling splitting of 0.79 cm^–1^ (24 GHz),^204^ as depicted in Figure 8, which gave rise to the first maser (the microwave early version of the laser).

**Figure 8:**
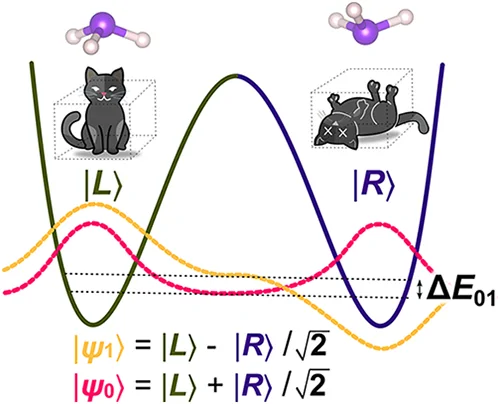
Coherent nuclear tunneling in a symmetric double-well potential illustrated for the ammonia umbrella inversion, depicting the |*L*⟩ and |*R*⟩ localized configurations, and the symmetric |ψ_0_⟩ and antisymmetric |ψ_1_⟩ delocalized eigenstates extending across both wells. The energy difference Δ*E*_01_ is the tunneling splitting between these two states.

A coherent state like this can be prepared only when there are no collisions with the environment (the “bath”) that can make it decohere. If the molecule can be maintained as a delocalized wavepacket, it makes no sense to speak in terms of localized tunneling from one well to another (which in chemical terms can be expressed as |*L*⟩ ↔ |*R*⟩ for the delocalized, and as |*L*⟩ ⇆ |*R*⟩ for the localized QT). However, its density (the probability of finding the system on the left or on the right after measurement) may still coherently evolve between the two wells with a frequency derived from the splitting energy).^149,205^

8 $$\nu_{t}=2\Delta E_{01}/h$$

This regime typically emerges when the barrier separating the wells is sufficiently low and narrow, and when interactions with the bath are weak enough to preserve phase coherence. Small perturbations that break symmetry reduce the tunneling coupling and progressively recover the localized regime characteristic of the previous section of this article,^64,104,149,206^ highlighting the nested structure of the matryoshka hierarchy. Nevertheless, the two regimes can be studied with different computational or experimental techniques, thus coherent QT does not really demand a previous localized QT analysis (although there are formulas that can obtain one from the other, as explained below).^183,187,189,207^

#### 3.3.1 “Real-Life” Experimental Observables

Coherent effects are mostly studied in physical systems, especially with photons (such as in the double-slit experiment, the “archetypal manifestation of coherence”,^64^ or with polarization studies), or with electrons (quantum transport, spin, etc.). The whole concept of quantum coherence as the correlation of wave functions is substantial and complex, and poorly defined.^64^ However, in here we are only focusing on cases of molecular quantum coherent tunneling.

As explained above, the primary observable associated with coherent tunneling is the Δ*E*_01_ between the two lowest nuclear energy levels, which can be directly determined from high-resolution spectroscopic measurements. These include microwave spectroscopy,^208–210^ rovibrational infrared spectroscopy,^211–213^ far-infrared spectroscopy,^214^ inelastic neutron scattering,^215,216^ and low-temperature nuclear magnetic resonance.^217^

Historically, several paradigmatic molecular systems have established Δ*E*_01_ as experimentally accessible quantities. The left–right superposition required for significant coherent QT comes, similar to the localized QT, from low barriers, low reduced mass of the reacting atoms, and especially narrow barriers. Thus, beyond the prototypical ammonia umbrella inversion introduced above, intramolecular proton-transfer systems such as malonaldehyde^218^ present large splittings on the order of 650 GHz,^211^ reflecting the light effective mass associated with hydrogen motion. Double proton transfer systems, including the formic acid dimer, also may display large splittings, in the range of 340 MHz,^219^ due to the short collective motion of multiple nuclei along the tunneling coordinate. Water clusters also exhibit complex tunneling splittings associated with hydrogen-bond rearrangements, typically ranging from kHz to MHz depending on the specific tunneling pathway.^220^ Due to their very low barrier, some conformational changes can also exhibit significant splitting, as in the case of phenol and hydrogen peroxide internal rotations;^221,222^ sometimes even heavy atom coherent QT can be observed in this type of simple reactions.^210^ Several examples of measured systems are presented in Figure 9. Interestingly, the tunneling splitting can be enhanced at excited vibrational states because of the lower and narrower effective barrier. And sometimes excited electronic states can generate the distorted geometries with double-well potentials that may present coherent tunneling on inactive molecules at their ground state.^213^ Isotopic substitution also provides important information, since the tunneling splittings strongly depend on the effective mass along the tunneling coordinate.^208^

**Figure 9:**
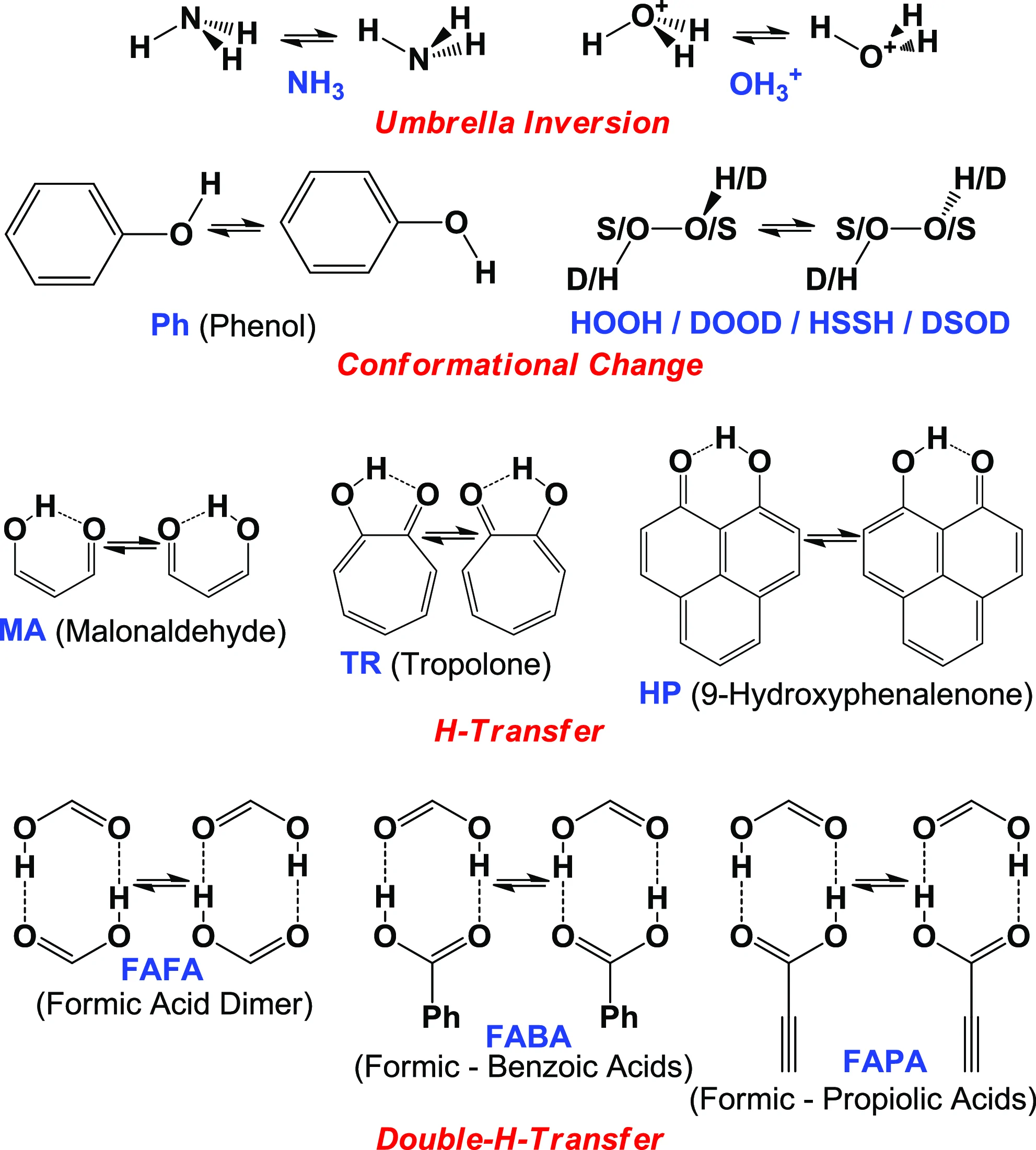
Some symmetric double-well chemical reactions whose tunneling splittings (Δ*E*_01_) have been accurately measured with spectroscopic techniques. Reproduced with permission from ref 183. Copyright 2025 Elsevier.

In some systems, coherent QT may also manifest as quantum beat signals in the time domain,^223^ providing a time-domain signature complementary to the Δ*E*_01_ splitting. When coherent superpositions of nuclear eigenstates can be prepared, time-domain spectroscopic methods such as ultrafast pump–probe spectroscopy, time-resolved infrared spectroscopy (TR-IR),^224^ and multidimensional vibrational techniques such as two-dimensional infrared spectroscopy (2DIR),^225^ and 2DES in systems where nuclear motion is strongly coupled to optically active electronic transitions,^226^ can reveal the oscillatory interference patterns arising from coherent superpositions. However, in most molecular systems coherent tunneling is primarily identified through high-resolution frequency-domain measurements of Δ*E*_01_ rather than direct observation of population oscillations in the time domain.

#### 3.3.2 Computational Frameworks

The theoretical description of coherent tunneling requires an explicit and accurate treatment of the quantum nuclear motion on the multidimensional PES exhibiting the double-well structure. QT depends, in the end, on the same barrier properties entering the semiclassical tunneling probability described in eq 4. After obtaining the multidimensional PES, several methods follow by solving nuclear Schrödinger equation using quantum vibrational methods over this surface. Variational approaches such as discrete variable representation (DVR),^213,227^ vibrational configuration interaction (VCI), and vibrational self-consistent field (VSCF)^228^ allow direct calculation of tunneling splittings through variational solutions, although an accurate description of tunneling typically requires inclusion of mode coupling beyond mean-field VSCF. Wavepacket-based approaches, including the multiconfigurational time-dependent Hartree (MCTDH) method,^229^ enable explicit treatment of coherent nuclear dynamics and multidimensional mode coupling. Diffusion Monte Carlo (DMC)^230^ methods offer an alternative stochastic approach for obtaining accurate ground-state splittings in strongly anharmonic or high-dimensional systems.

In addition, descriptions arise from semiclassical-QT and path-integral frameworks, where tunneling is represented in terms of imaginary-time trajectories connecting symmetry-related minima. Instanton theory and its path-integral implementations, such as ring-polymer instanton (RPI) approaches,^181,207,231^ allow practical semiclassical estimates of tunneling splittings in multidimensional systems, particularly in the deep tunneling regime. PIMD^232^ provides an alternative framework for incorporating nuclear quantum delocalization and zero-point fluctuations in complex potential energy landscapes, while WKB-type approaches yield simplified one-dimensional estimates of Δ*E*_01_ based on the semiclassical-QT action.^233^

Connections between localized and delocalized descriptions of tunneling provide an additional route for estimating Δ*E*_01_. As explained above, even if they involve a different kind of physics, both coherent tunneling splittings and localized rate constants depend on the same semiclassical action (θ, see eq 4), thus being logical that a formula may convert one from the other. There was a confusion in the literature regarding this formula, with some sources considering the linear trend of eq 8 (*k* ∝ Δ*E*_01_), whereas others postulated an approximate quadratic relationship (*k* ∝ Δ*E*_01_^2^).^183,207,234,235^ The most advanced mathematical analysis and recent semiempirical relationships using SCT and RPI^183,207^ indicate that the latter is correct. The misunderstanding is just a misinterpretation of physical concepts: for localized QT in a double-well, the rate of the molecule going from one state to the other is quadratic; but for coherent QT, the probability of finding the system in one or the other state upon measurement fluctuates in a linear fashion (eq 4). Despite being two different concepts, our estimations^183^ indicate that we can translate from one to the other according to

9 $$k\approx \frac{e\pi }{h^{2}\nu_{R}}\Delta E_{01}^{2}$$

with ν_*R*_ being the frequency of the normal mode leading to the reaction at the stationary state. This provides a practical estimate when a direct solution of the nuclear Schrödinger equation becomes computationally demanding, establishing a conceptual bridge between this and the previous levels. This idea has been applied in several hydrogen transfer reactions. In a more recent example, a fast QT in π-bond shifting in pentalene-based frameworks was also deemed to potentially produce coherent tunneling, which can modulate the local aromaticity character, theoretically making the rings both aromatic and antiaromatic at the same time; this was called a “Schrödinger’s aromaticity cat.”^187^

#### 3.3.3 Remarks for the Hierarchy

These last two levels provide alternative regimes of nuclear tunneling that differ in the facet of the nuclear motion: a localized rate process of chemical nature, versus delocalized nuclear eigenstates giving rise to tunneling splittings. Both regimes originate from the same barrier penetration mechanism and depend on similar physical parameters, including barrier height, width, and effective mass. However, the quantum superposition in the latter may encourage us to consider it a “higher level” of quantum system. Nevertheless, environmental perturbations with the bath may easily suppress coherence, leaving a “lesser” quantum system only capable of performing a back and forth localized tunneling.

One last remark on this level is the fact that coherent QT is the core of the growing field of quantum computing.^236^ A system that can maintain coherence between two states in superposition, and its state can be measured at some point, can be, in principle, made into a qubit. In practice, this has been done tinkering with the phase of electronic superconductors on Josephson junctions,^237^ with the spin state in quantum dots,^238^ with the trapping of ions or atoms having two accessible electronic states, with the polarization of photons, or with other physical methods. The discussion on this topic and the advantages and disadvantages of these techniques is left to the specialists, which we are not. However, we can still wonder if coherent quantum tunneling of chemical systems may have a future place in this enigmatic field.

## 4 Quantum Subatomic Particles (Beyond the Electron!)

In principle, there are deeper levels of quantum description beyond the electron–nucleus Hamiltonian commonly used in chemistry and molecular physics. Evidently, the next level will involve the nucleons: protons and neutrons. Beyond it, and neglecting elementary bosons like the photon, matter can be described in terms of hadrons (combinations of quarks) and leptons (electrons, muons, tauons, and neutrinos), that is, the “standard model of particle physics”. The interactions governing them are formulated within quantum field theories, such as quantum electrodynamics (QED) and quantum chromodynamics (QCD), which pertain more to nuclear and particle physics than to molecular chemistry.

Maybe we will never know how many other levels there are in the matryoshka, or if there is a limit to how much can we cut matter into smaller entities (paraphrasing Democritus, who coined the term atom, from the Greek ατομον, “uncuttable”, Figure 10). This is one of the big ontological questions, if not the biggest. In any case, this is a discussion way outside the realm of chemistry.

**Figure 10:**
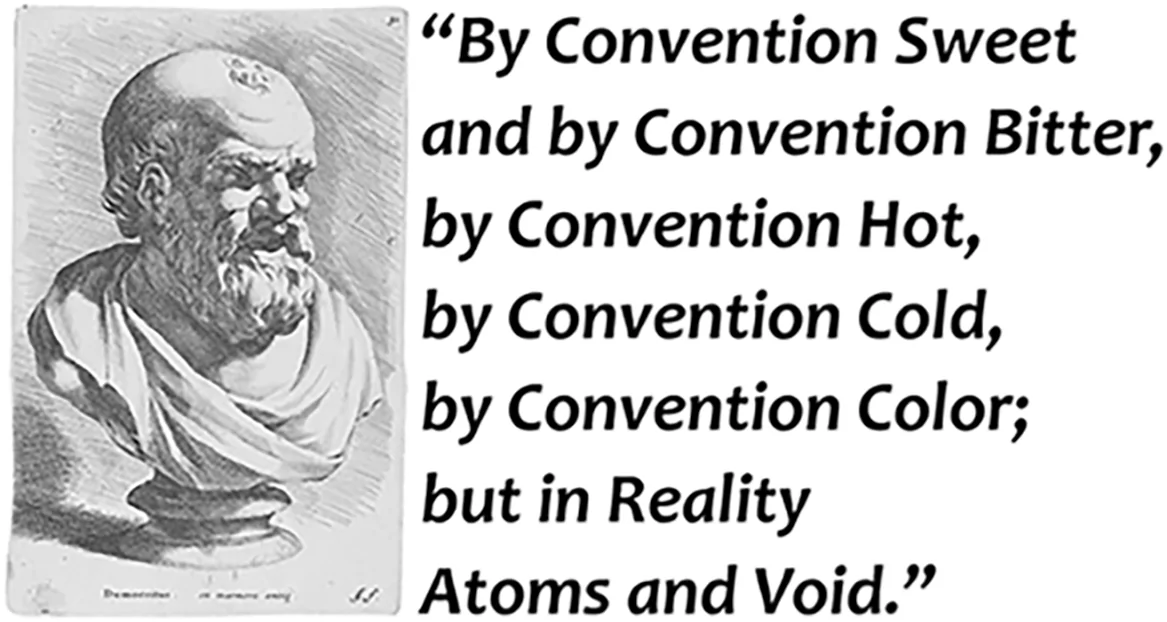
Democritus, the “laughing philosopher”, and his most famous quote on atomism, indicating that the “uncuttable” particles only have shape and size qualities.^239^ Two millennia later, Dalton proposed that these indivisible particles are what we now know as atoms, and two centuries after him we are still unsure if Democritus was right. Portrait adapted with permission from ref 240.

Among the characteristic phenomena at this level of quantum subatomic particles are nuclear decay, processes mediated by the weak interaction, and exotic systems such as muonic atoms.^241^ These processes occur at energy scales on the order of MeV, far above the eV range characteristic of chemical bonding, molecular spectroscopy, and the nuclear quantum effects discussed in the previous levels.^242^ Although these interactions underlie the ultimate structure of matter, in molecular chemistry their effects are effectively incorporated into constant parameters such as nuclear masses, charges, and decaying rates in radiochemistry (essentially being taken as emergent properties). Consequently, they do not significantly modify PES, reaction barriers, or the energy splitting in any appreciable way, falling outside the theoretical framework normally required to describe molecular structure and chemical reactivity. Nevertheless, their existence reminds us that the hierarchy of quantum descriptions extends beyond the electron–nucleus model, reinforcing the layered organization that inspires the matryoshka-type analogy.

However, there are weird exceptions. We can cite here, as a unique and extreme example, the exotic case of muonic chemistry, in which a proton is replaced by an antimuon (μ^+^), a lepton with one-ninth of the mass of a hydrogen, and a lifetime of a couple of microseconds. Evidently, such systems can produce very large tunneling rates and KIEs,^243^ which has been proven computationally and experimentally.

## 5 Conclusions

The matryoshka analogy does not suggest that each level requires a different approach, nor that every quantum effect must necessarily be included before considering the next one, but rather describes the progressive inclusion of additional quantum effects within the same fundamental problem. Moreover, since the analogy is for the eyes of the chemists, it is not arranged as a function of particle masses but as a function of particle importance for chemical processes (thus the lightest particles are at the innermost and outermost layers). In principle, all these phenomena are contained within the complete quantum description of the molecular system; what changes between levels are which degrees of freedom are explicitly treated as quantum and which approximations are appropriate for the type of chemistry of interest. A particular quantum effect needs to be unlocked only when the system mass, energy, or time scales probed by the experiment are comparable to the characteristic scale of the process. For example, vibrational quantization is necessary to interpret IR spectra, while the tunneling effect of heavy atoms can be essential to explain anomalous rate constants or KIEs outside the classical regime. In this sense, this analysis does not separate chemical systems into categories, but rather into hierarchies, levels of description that may be necessary to interpret depending on the studied observables. Still, there are branching possibilities

As a general, rather obvious rule in quantum computational chemistry, and as already expressed above, ignoring a level can lead not only to quantitative errors but also to qualitatively incorrect chemical and physical conclusions. For example, omitting the ZPE can significantly alter the effective barrier height, or neglecting the tunneling effect can lead to the conclusion that a molecule is kinetically stable when it may actually have a short lifetime. Again, these are effects that cannot be switched off in the real-world experiments, so they must be judicially turned on in the computational experiment. It is always wise to remember that we do not know a model until we know its limits, and this is especially important for computational chemistry. It comes with the job.

From a more philosophical perspective, this brings up the debate of reductionism versus emergence.^244^ The former is defined as the possibility of understanding one theory from a more basal, maybe more encompassing one (typically exemplified with the reduction of biology into chemistry, and this into physics). Emergence, on the contrary, implies that complex effects cannot be predicted from the more fundamental theories (biology being an “emergent property” of chemistry which cannot be reduced to it, and chemistry similarly emerging from physics without being encompassed by it). Essentially, we do not require quantum chromodynamics and the standard model of particles to understand the chemical bond; this chemical concept can, on paper, be derived (“reduced”) from these more fundamental models, but in practice it will be a futile endeavor, requiring enormous effort for something that raises (“emerges”) from a different dimension of complexity. But we surely need to understand the tenets of quantum mechanics!

Every scientist must find their niche, juggling between the two opposed concepts of reductionism and emergence in the philosophy of sciences. But in this case, what is natural and effortless for *in vitro* chemistry, is a skill that must be carefully learned for *in silico* chemists.

## §

Dedicated to the memory of Yitzhak Apeloig.
